# Characterization of Instrument Transformers under Realistic Conditions: Impact of Single and Combined Influence Quantities on Their Wideband Behavior

**DOI:** 10.3390/s23187833

**Published:** 2023-09-12

**Authors:** Palma Sara Letizia, Gabriella Crotti, Alessandro Mingotti, Roberto Tinarelli, Yeying Chen, Enrico Mohns, Mohamed Agazar, Daniela Istrate, Burak Ayhan, Hüseyin Çayci, Robert Stiegler

**Affiliations:** 1 Istituto Nazionale di Ricerca Metrologica (INRIM), Strada delle Cacce 91, 10135 Torino, Italy; g.crotti@inrim.it; 2 Department of Electrical, Electronic and Information Engineering, Guglielmo Marconi, Alma Mater Studiorum, University of Bologna, Viale del Risorgimento 2, 40136 Bologna, Italy; alessandro.mingotti2@unibo.it (A.M.); roberto.tinarelli3@unibo.it (R.T.); 3 Department 2.3-Electrical Energy Measuring Techniques, Physikalisch-Technische Bundesanstalt (PTB), Bundesallee 100, 38116 Braunschweig, Germany; yeying.chen@ptb.de (Y.C.); enrico.mohns@ptb.de (E.M.); 4 Laboratoire National de Métrologie et d’Essais (LNE), 1 Rue Gaston Boissier, 75015 Paris, France; mohamed.agazar@lne.fr (M.A.); daniela.istrate@lne.fr (D.I.); 5 Power and Energy Laboratory, TÜBİTAK National Metrology Institute, 41470 Gebze, Kocaeli, Türkiye; burak.ayhan@tubitak.gov.tr (B.A.); huseyin.cayci@tubitak.gov.tr (H.Ç.); 6 Institute of Electrical Power Systems and High Voltage Engineering, TUD Dresden University of Technology, 01062 Dresden, Germany; robert.stiegler@tu-dresden.de

**Keywords:** instrument transformers, distribution grids, accuracy, influence quantity, power quality, harmonics, measurement traceability

## Abstract

Instrument transformers (ITs) play a key role in electrical power systems, facilitating the accurate monitoring and measurement of electrical quantities. They are essential for measurement, protection, and metering in transmission and distribution grids and accurately reducing the grid voltage and current for low-voltage input instrumentation. With the increase in renewable energy sources, electronic converters, and electric vehicles connected to power grids, ITs now face challenging distorted conditions that differ from the nominal ones. The study presented in this paper is a collaborative work between national metrology institutes and universities that analyzes IT performance in measuring distorted voltages and currents in medium-voltage grids under realistic conditions. Both current and voltage measuring transformers are examined, considering influence quantities like the temperature, mechanical vibration, burden, adjacent phases, and proximity effects. The study provides detailed insights into measurement setups and procedures, and it quantifies potential errors arising from IT behavior in measuring distorted signals in the presence of the various considered influence quantities and their combinations. The main findings reveal that the temperature has the most evident impact on the inductive voltage transformer performance, as well as the burden, causing significant changes in ratio error and phase displacement at the lower temperatures. As for low-power ITs, establishing a priori the effects of adjacent phases and proximity on the frequency responses of low-power ITs is a complex matter, because of their different characteristics and construction solutions.

## 1. Introduction

To achieve the net-zero greenhouse gas emissions target set by the European Union by 2050 [1], the use of renewable energy sources and electric mobility is significantly increasing. This transition implies more power converters to connect power sources and energy storage systems to the electrical grid. However, power converters introduce disturbances into the grid due to their frequency switching [2], deteriorating the power quality (PQ). Thus, monitoring PQ is crucial in modern power systems. At the medium voltage (MV) level, the PQ measurement chain always includes instrument transformers (ITs) upstream of the low-voltage (LV) PQ analyzers.

ITs are then a key component in electrical power systems, to enable the accurate and safe measurement and monitoring of electrical quantities [3]. These devices are designed to transform the grid’s high currents and voltages into lower levels suitable for the LV input of measurement instrumentation and protection devices. Data obtained from measurement chains that include ITs are used for various critical functions, including power system protection, metering for billing and revenue purposes, system control for efficient grid management, and, as highlighted previously, PQ assessment.

Nowadays, ITs built with different operating principles can be found in MV grids. Traditionally, MV ITs based on the principle of electromagnetic induction have been adopted to reduce the grid voltage and current to lower levels. These ITs are reliable and stable, but they suffer from iron core non-linearity, and, due to stray capacitance, their frequency responses can exhibit resonances in the harmonic range depending on the primary voltages. As a consequence, the frequency range in which they can accurately operate may be limited with respect to the frequency spectrum of the PQ disturbances to be measured. In the last few decades, new ITs, namely low-power (LP) ITs, have been introduced, based on operating principles such as voltage dividers for voltage sensors and the air-core, toroidal-core, or zero-flux principle for current sensors. They can be active or passive and can provide analogue or digital outputs. An in-depth analysis of the different ITs and their operating principles is provided by the Technical Report (TR) IEC 61869-103 [4].

The general prescription concerning requirements to be satisfied by ITs is well detailed in the IEC 61869 series, which is entirely dedicated to ITs. In detail, IEC 61869-1 [5] and -6 [6] are dedicated to the general requirements for ITs and LPITs, respectively. IEC 61869-2 [7], -3 [8], and -4 [9] deal with inductive current transformers (CTs), inductive voltage transformers (VTs), and combined ITs, respectively. Analogously, IEC 61869-10 [10] and -11 [11] deal with the LP current and voltage transformers (LPCTs and LPVTs). Each standard includes suggestions for manufacturers and users, test procedures for the evaluation of the ITs’ performance, and relevant accuracy limits, rated quantities, and so on.

As for the present research on ITs, it is highly developed and up to date [12]. For example, the calibration of VTs/CTs, digital ITs, and non-conventional ITs is treated in many papers [13,14,15,16,17,18,19,20,21,22]. The proposal of new testing techniques is discussed in [23], while refs. [24,25] present predictive maintenance approaches for ITs and CTs. Design techniques and new construction solutions are described in [26,27]. Finally, a modelling solution for LPCTs and compensation methods for electronic ITs are detailed in [28,29], respectively.

The work presented in the following aims to experimentally assess the impact of various influence quantities on the wideband performance of ITs. The paper provides a description of innovative traceable generation and measurement setups specifically designed, realized, and characterized by national metrology institutes (NMIs) and universities, to characterize ITs in conditions representative of the realistic MV ones, i.e., considering MV-level distorted waveforms with spectrum content from power frequency up to 9 kHz. In addition, influence quantities like temperature, vibrations, burden, the electric and magnetic fields produced by adjacent phases, and proximity effects due to grounded structures are reproduced in the laboratory. The proposed study covers different types of commercial ITs and quantifies the effects of the influence quantities both individually and in a combined manner, focusing on the assessment of the frequency responses of the sensors.

To the best of the authors’ knowledge, this topic is quite new and not deeply studied yet. In particular, from the standardization point of view, the IT IEC 61869 standard family does not provide indications regarding the quantification of the influence quantities’ impacts on the frequency responses of ITs. In particular, for some influence quantities and for a subset of IT types, the IEC 61869 standard only requires one to evaluate the effects on the power frequency ratio error and phase displacement. As regards the scientific literature, a number of papers dealing with the assessment of the influence quantities on ITs’ wideband responses can be found. However, these published papers only refer to certain influence quantities and investigate specific types of ITs [30,31].

Current and voltage sensors’ frequency performance can be investigated numerically and/or by circuital models (see, e.g., [32,33]), but such a type of analysis relies on detailed knowledge of the considered sensors and is not feasible in the case of commercial sensors of different types, whose construction details are not easily made available.

A comprehensive investigation of the influence quantities’ effects on ITs’ wideband performance is presented in the following, to contribute to addressing the existing gap in both the standards and scientific literature.

The activity described in the paper is part of the European Project “Measurement methods and test procedures for assessing the accuracy of *I*nstrument *T*ransformers *f*or *P*ower *Q*uality measurements (IT4PQ) [34]. The project was developed within the European Metrology for Innovation and Research Programme (EMPIR), with the primary objective of setting up a metrological framework that enables the traceable calibration of ITs for use in PQ measurements in electricity distribution grids.

The specific technical issues faced are the following.

i. The definition of the accuracy and uncertainty limits of ITs in PQ measurements, functional to the establishment of “PQ Accuracy Classes” as an extension of the power frequency accuracy class concept of ITs to the frequency range up to 9 kHz. To this end, significant test waveforms and suitable performance indices for PQ parameters have been defined and experimented, as detailed in [35].
ii. The development of missing reference measurement systems and related test procedures and methodologies to evaluate the relevant uncertainty contribution of ITs to PQ indices, to ensure the traceability and accuracy of the measurement results provided of ITs in the wideband measurement of PQ disturbances. On this subject, reference systems for the laboratory frequency characterization of VT/LPVT and CT/LPCT have been developed starting from previous experience. An example of a realized setup and its characterization is given in [16].
iii. The implementation of simplified IT test procedures, which can be easily adopted by test laboratories that operate in an industrial environment. To this end, simplified but accurate procedures to characterize wideband ITs have been studied and validated by comparison with the reference ones as implemented by NMIs. An example of a procedure for VTs’ wideband characterization that makes use of measurement instrumentation commonly used in industrial laboratories is described in [36].
iv. The evaluation of ITs’ performance under realistic conditions, including the impact of the simultaneous presence of different influence quantities. The adopted procedure, systems, and findings of the activity carried out on this last topic are the subject of the present paper, as described in the following sections, considering the previously mentioned influence quantities.

Outputs from all these activities are expected to benefit both the scientific and technical communities, as well as to be a useful input to the relevant standardization work.

**Figure 1:**
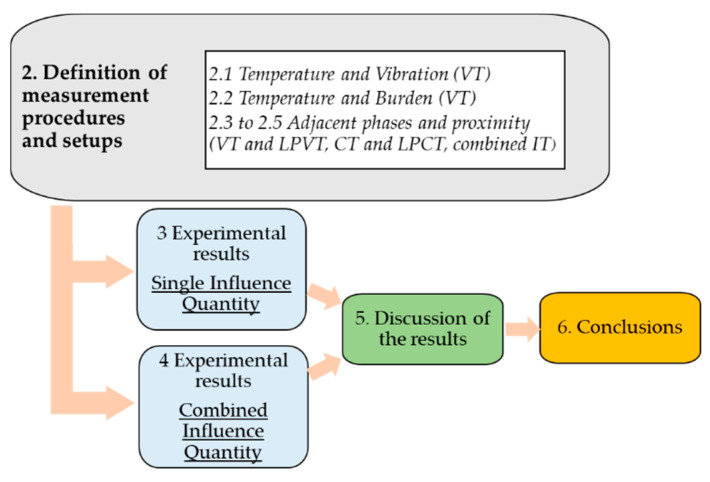
Graphical representation of the paper’s content.

The paper is structured as depicted in Figure 1. In particular, in Section 2, all measurement setups and tests performed on ITs are comprehensively described, whereas Section 3 presents a detailed description of the obtained results in the presence of a single influence quantity. Section 4 provides experimental results under combined influence quantities. Finally, Section 5 and Section 6 provide an in-depth analysis of the results and a comprehensive conclusion, respectively.

## 2. Measurement Procedures and Setups

The measurement setups are described in the following, along with the test procedure adopted for the investigation of IT performance at power frequency and up to 9 kHz. In detail, each subsection is dedicated to the setup and procedure for one or more influence quantity considering temperature, vibrations, electric and magnetic fields generated by adjacent phases, proximity effects of grounded metallic structures, and IT burden. Specific solutions adopted are dealt with as a function of the different types of investigated ITs (VT and CT, LPVT and LPCT, combined current and voltage ITs). For each IT and considered influence quantity, the activity is developed according to the following steps.

Step 1: Identification of the range of variation of the influence quantity.

Step 2: Definition of the test waveforms and, in particular, of the applied harmonics in terms of frequencies and amplitudes.

Step 3: Test of the Device Under Test (DUT) under waveforms as defined in Step 2. in the presence of a single influence quantity, varying it within the defined range.

Step 4: For each test waveform and condition (Step 3), evaluation of the harmonic performance indices (PIs), defined in terms of the ratio and phase errors at fundamental at harmonic tones by [35]

(1) $$\varepsilon \left(f_{n}\right)=\frac{k_{r}\cdot Q_{s}\left(f_{n}\right)-Q_{p}\left(f_{n}\right)}{Q_{p}\left(f_{n}\right)}$$

(2) $$\varphi \left(f_{n}\right)=\varphi_{s}\left(f_{n}\right)-\varphi_{p}\left(f_{n}\right)$$

where 

$k_{r}=\frac{Q_{p,r}}{Q_{s,r}}$ is the IT’s rated transformation ratio; $f_{n}$ is the frequency of the applied tone from 50 Hz (*f*_0_) to the highest investigated harmonic frequency; $Q_{p}\left(f_{n}\right)$ and $Q_{s}\left(f_{n}\right)$ are the values of the primary and secondary quantities (current or voltage), respectively, at the frequency $f_{n}$; $\varphi \left(f_{n}\right)$ is the phase displacement at $f_{n}$ between the phases $\varphi_{s}\left(f_{n}\right)$ and $\varphi_{p}\left(f_{n}\right)$ of the secondary and primary quantity phasors, respectively. The phase displacement is assumed to be positive when the secondary quantity phasor leads the primary quantity one.

Step 5: Test of the DUT in the presence of two influence quantities, varying both of them to investigate all possible configurations.

Step 6: For each test performed in Step 5, evaluation of the PIs for each frequency tone $f_{n}$, as defined in Equations (1) and (2).

Step 7: Comparison and analysis of the PIs measured in Step 4 and Step 6.

### 2.1. Influence of Temperature and Vibration on VTs

#### 2.1.1. Introduction

Mechanical vibrations can have detrimental effects on the integrity of ITs, leading to the displacement of the windings and the core or causing the separation and compression of turns. These internal mechanical changes may impact the stray capacitances of the IT, subsequently affecting its frequency response. In certain cases, vibrations generate friction on the magnetic core, influencing its magnetizing inductance, accelerating ageing, and ultimately reducing its accuracy. Vibrations can have a significant impact, contributing to winding displacement and deformation. For instance, axial vibrations can separate and compress the turns, radial vibrations may cause turn displacement around the core, and vectorial vibrations can lead to winding deformations. Temperature also plays a critical role in IT performance. The electrical and magnetic properties of all IT components are subject to changes with temperature. Passive components like resistances, capacitances, and inductances are temperature-dependent. Moreover, the permeability of the magnetic core and the permittivity of insulating materials are influenced by temperature changes.

#### 2.1.2. Measurement Setup

**Figure 2:**
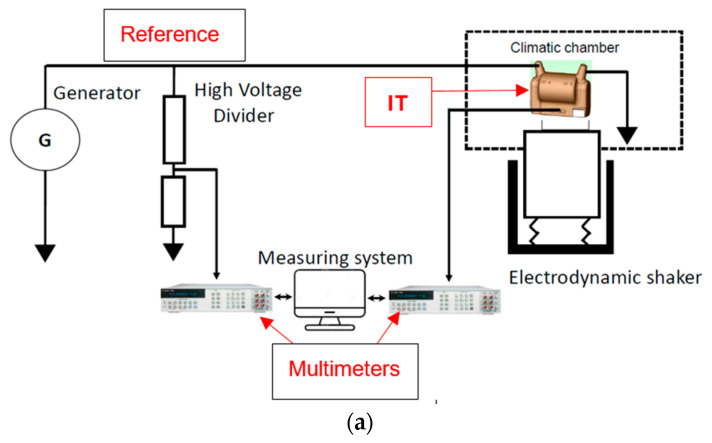

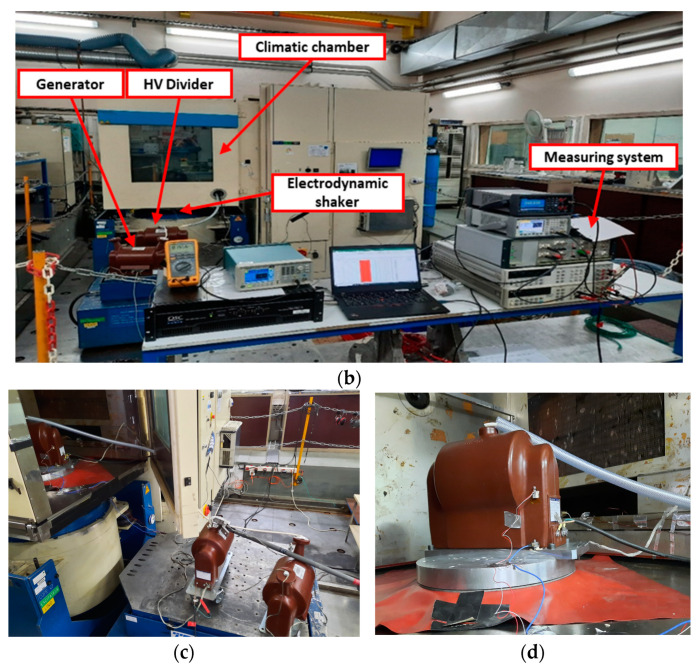
Assessing VT frequency response under temperature and vibration. (**a**) Scheme of the measuring system; (**b**) overview of the whole setup arranged at LNE; (**c**) detail of the generator and the voltage standard transformer, (**d**) DUT mounted onto the shaker inside the temperature chamber.

The Laboratoire National de Métrologie et d’Essais (LNE) has developed a comprehensive setup for the accurate characterization of the frequency responses of VTs under the influence of both temperature and vibration [31]. The test circuit of this setup is illustrated in Figure 2. The measuring system comprises a high-voltage standard divider in conjunction with a low-voltage measuring system (LVMS). The LVMS is used to precisely measure the ratio error and phase displacement between the output voltages of both the reference high-voltage divider and the transformer being tested. It utilizes two sampling voltmeters, which are recognized as analogue-to-digital converters for accurate AC measurement [37,38]. The uncertainties associated with the measurements of the ratio error and phase displacement provided by the whole measuring system are numerically expressed as a function of the frequency *f* as $\left(0.13\cdot f+20\right)$ µV/V for the ratio error and $\left(0.13\cdot f+20\right)$ µrad for the phase displacement. These highly accurate measurements enable the comprehensive assessment of the VT performance under the combined influence of temperature and vibration.

**Figure 3:**
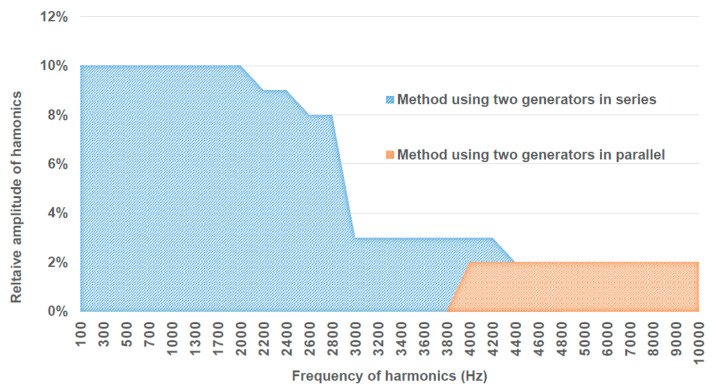
LNE generation setup: harmonic amplitude generation capabilities expressed as % of the fundamental tone for the two generation methods implemented.

The setup also includes two voltage generation systems capable of producing 30 kV at rated power frequency with a superimposed harmonic tone up to 9 kHz and beyond. The level of harmonics varies with frequency, with amplitudes relative to the fundamental tone from 10% at 100 Hz to approximately 2% at 9 kHz, as depicted in Figure 3, which gives harmonic amplitude generation capabilities expressed as a percentage of the fundamental tone for the two generation methods implemented.

The first generator utilizes two voltage transformers arranged in series, where one transformer generates the fundamental amplitude, and the second one generates the harmonic amplitude. However, the performance of this first generator is constrained by the current consumption at higher frequencies. In contrast, the second generator is a parallel arrangement of two grounded voltage sources: a voltage transformer to generate the fundamental frequency and a high-frequency power amplifier to produce harmonics up to 9 kHz and beyond. Suitable blocking elements are incorporated to protect one source from interference with the other. Finally, the setup includes an electrodynamic shaker to generate vibrations. The shaker is capable of vibrating in the vertical, longitudinal, and horizontal directions, with a vibration range from 3 Hz to 2 kHz. The peak-to-peak displacement can be configured between 25 mm and 75 mm. To facilitate vertical vibration tests, the climatic chamber was modified to accommodate the VT securely over the electromagnetic shaker.

#### 2.1.3. Test Procedure

A 1% accuracy class inductive VT was tested to assess the impact of vibration and temperature both individually and in a combined manner on its accuracy in performance. The DUT was designed to operate within the temperature range of −25 °C to +55 °C selected for the testing. The tests were conducted with a resistive burden of 1 VA. Before each series of tests, the measurement setup was calibrated, and the necessary corrections were applied to ensure the highest accuracy. The first step of the analysis was the evaluation of the single influence quantity effect, and, subsequently, the results under combined temperature and vibration were obtained with reference to the VT ratio error and phase displacement both at power frequency and up to 4 kHz.

The temperature classes for ITs are specified in [6] and in IEC 60721-3-3 [39]. The LNE test platform allows for testing with the largest interval. The applied temperature profile followed the indications given in [6], with three selected temperatures: −25 °C, 23 °C, and 55 °C. Regarding vibration, most vibration phenomena encountered in civil applications (e.g., seismic, road traffic, railway traffic, power transformers, wind, rain, switching devices, etc.) do not exceed 150 Hz. To cover a wide range of possibilities, the vibration frequency was varied from 3 Hz to 150 Hz (specifically, 3 Hz, 20 Hz, 100 Hz, and 150 Hz), and the acceleration of vibration was set at 0.1 g, 0.3 g, and 0.5 g (where g ≈ 9.81 m/s^2^).

The tests were conducted along three axes, namely the vertical (Z), horizontal longitudinal (X), and transversal (Y) directions. To examine the frequency response of the VT under combined temperature and vibration quantities, a low-level, high-frequency sinusoidal voltage was superimposed on the 50 Hz high voltage.

The amplitude of voltages from 150 Hz to 2000 Hz was set at 5% of the nominal voltage of the tested VT. For higher frequencies up to 4000 Hz, the amplitude was generated with 3% of the nominal voltage.

The entire measurement procedure, which consists of two main steps (A and B), is summarized in the following.

A. VT performance assessment under separate temperature and vibration
  a. Setup of the shaker at *Z* axis.
  b. Measurement of the VT PIs at fundamental frequency without vibration for all three temperatures (−25 °C, 23 °C, and 55 °C).
  c. Measurement of the VT PIs under vibration for all three temperatures (−25 °C, 23 °C, and 55 °C), all vibration frequencies (3 Hz, 20 Hz, 100 Hz, and 150 Hz), and all accelerations (0.1 g, 0.3 g, and 0.5 g).
  d. Setup of the shaker at *X* axis.
  e. Measurement of the VT PIs in the absence of vibration at 23 °C.
  f. Measurement of the VT PIs at 23 °C under vibration for all vibration frequencies (3 Hz, 20 Hz, 100 Hz, and 150 Hz) and all accelerations (0.1 g, 0.3 g, and 0.5 g).
  g. Setup of the shaker at the *Y* axis and at 23 °C.
  h. Measurement of the VT PIs without vibration at 23 °C.
  i. Measurement of the VT PIs at 23 °C under vibration for all vibration frequencies (3 Hz, 20 Hz, 100 Hz, and 150 Hz) and all accelerations (0.1 g, 0.3 g, and 0.5 g).

B. Assessment of VT frequency response under temperature and vibration conditions.
  a. Setup of the shaker at the *Z* axis.
  b. Measurement of the VT’s frequency response without vibration for all three temperatures.
  c. Measurement of the frequency response of the VT under vibration for all three temperatures (−25 °C, 23 °C, and 55 °C) under vibration frequencies of 20 Hz and 100 Hz at 0.5 g acceleration.

### 2.2. Influence of Temperature and Burden on VTs

#### 2.2.1. Generation and Measurement Setup

**Figure 4:**
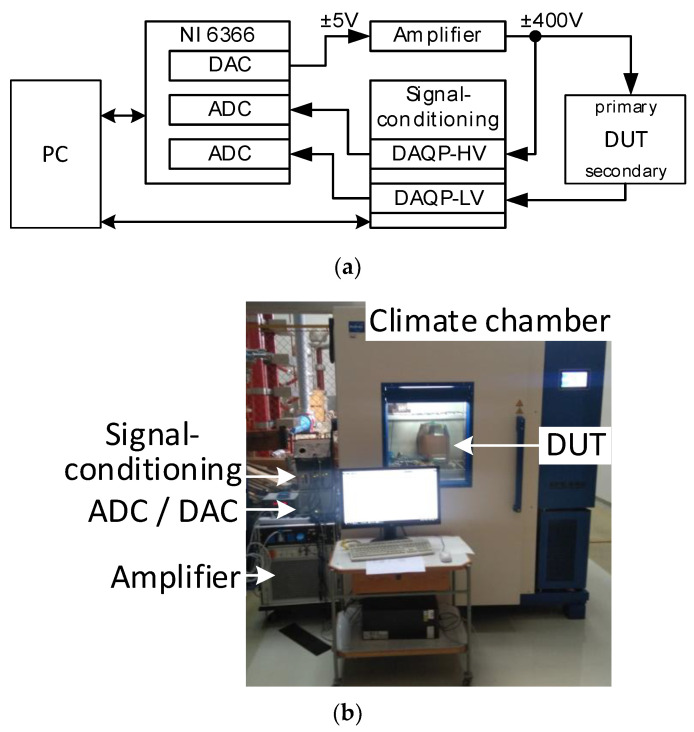
Setup for the measurement of the frequency responses of VTs at TU-Dresden: (**a**) block scheme; (**b**) picture of the setup.

The investigation of the separate and combined effects of the temperature and burden was performed in the TU-Dresden laboratories. The measurement setup used for the determination of the burden and temperature’s impact on the frequency behavior of VTs is shown in Figure 4. The DUTs were placed in a climate chamber; the measurement setup and additional burden were outside the climate chamber. Since a realistic measurement at the rated voltage of the DUT could not be used in the climate chamber, a simplified setup was used. The fundamental and higher-frequency components were generated together with one DAC channel of a multifunction device, the NI 6366. The signal was amplified with a broadband amplifier. The primary and secondary voltages of the DUT were conditioned with Dewetron DAQP-HV and DAQP-LV modules and measured with two ADC channels of the NI 6366 with a sampling rate of 600 kS/s.

#### 2.2.2. Test Procedure

Four MV VTs from different manufacturers and different rated primary voltages were tested (Table 1). As a preliminary step, the thermal time constant of each DUT was estimated with a temperature jump and the measurement of the secondary winding resistance.

For the combined influence of the burden and temperature on the frequency responses of the VTs, the four DUTs were placed in a climate chamber. After each change in the temperature, a settling time of at least ten times the time constant was adopted to ensure a thermally steady state. The temperature was changed in ten steps from −25 °C to 55 °C. At each temperature step, the burden was varied in 7 steps from 0% to 100% of the rated burden power.

For each DUT and each burden/temperature state (70 states), the frequency response from 50 Hz to 10 kHz was measured [30].

### 2.3. Influence of Adjacent Phases and Proximity on VTs and LPVTs

#### 2.3.1. Generation and Measurement Setup

**Figure 5:**
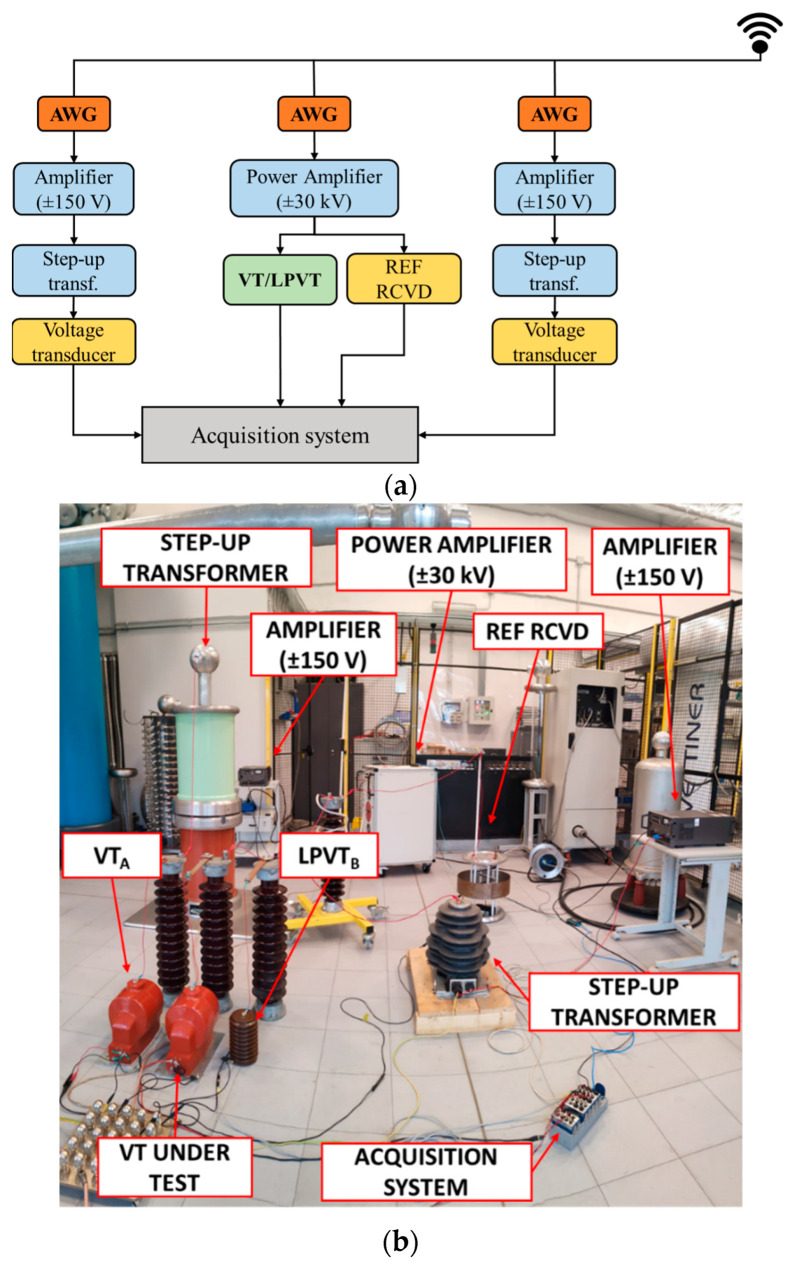
INRIM generation and measurement setup for the assessment of adjacent phases and proximity effects on VT/LPVT ratio error and phase displacement versus frequency: (**a**) block scheme; (**b**) setup arrangement in the INRIM HV laboratory.

A dedicated three-phase generation and measurement setup was developed at INRIM to assess the impact of adjacent phases and the effect of the proximity of grounded objects on both VTs and LPVTs used for wideband PQ measurement (Figure 5).

The LV generation was obtained by synchronizing three arbitrary waveform generators (AWG), the NI PXI (National Instruments PCI eXtension for Instrumentation) 5422, with a 16-bit, variable output in the ±12 V range, with a 200 MHz maximum sampling rate and 256 MB memory.

The MV signal was applied to the VT/LPVT under test using a Trek high-voltage power amplifier (±30 kV, ±20 mA) with a wide bandwidth (from DC to 2.5 kHz at full voltage and 30 kHz at reduced voltages). The two adjacent phase low-voltage supplies were amplified in two stages: first, by using two LV power amplifiers, NF HSA42014, with a ±150 V maximum output voltage, and by introducing two step-up transformers.

To measure the MV applied to the VT/LPVT under test, a ±30 kV wideband reference resistive–capacitive voltage divider (REF RCVD) was used. Moreover, two VTs or one VT and one LPVT (Figure 5, VT_A_ and LPVT_B_) were employed to measure the adjacent phase voltages. For the data acquisition, a compact DAQ chassis with multiple acquisition modules (ranging from ±0.5 V to ±425 V, 50 kHz maximum sampling frequency) was employed. The system was configured to acquire 4 different signals: the output of the REF RCVD, the one measured by the VT/LPVT under test, and those of the monitoring VT/LPVT. The uncertainty (level of confidence 95%) of the ratio errors and phase displacements up to 9 kHz was 200 µV/V and 300 µrad, respectively.

#### 2.3.2. Test Procedures

Four different commercial MV voltage sensors were tested: three LPVTs with different operating principles (a resistive divider, a resistive–capacitive divider, a pure capacitive divider) and one inductive VT. Their main characteristics are provided in Table 2.

- Adjacent Phase Test

**Figure 6:**
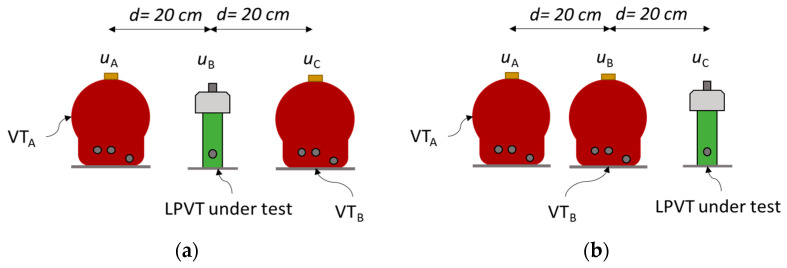
Testing with LPVT in (**a**) central position (OXO) and (**b**) external position (OOX).

Two configurations were considered for the VT/LPVT tests: one with the VT/LPVT in the central position (see LPVT example in Figure 6a, configuration “OXO”, where “X” represents the DUT and “O” indicates the adjacent phase voltage measuring devices), and one were where the VT/LPVT was on the external side (Figure 6b, configuration “OOX”). The distance between the central axes of the three transducers (VT_A_, VT_B_, and VT/LPVT under test) was 20 cm.

Three voltages were digitally synthesized to quantify the influence of the electric field produced by adjacent phases on the VT/LPVT measurement of harmonic voltages in two different cases: (i) when an in-phase power supply and (ii) when a three-phase power supply was applied. For the “in-phase” power supply, the two adjacent phases were supplied with sinewave voltages with same MV amplitude *V*_r_ = 20/√3 kV and the same phase of the fundamental component of the voltage applied to the VT/LPVT under test:

(3) $$v_{A}\left(t\right)=v_{B}\left(t\right)=v_{C}(t)\;=\;\sqrt{2}V_{r}sin\left(2\pi f_{n}t\right)$$

where *f*_n_ is the frequency of the fundamental tone (50 Hz).

When the three-phase supply case was considered, the three voltages had the same fundamental component amplitude *V*_r_, but with a phase delay of ±2π/3:

(4) $$v_{A}\left(t\right)=\sqrt{2}V_{r}sin\left(2\pi f_{n}t\right)$$

(5) $$v_{B}\left(t\right)=\sqrt{2}V_{r}sin\left(2\pi f_{n}t+\frac{2\pi }{3}\right)$$

(6) $$v_{C}\left(t\right)=\sqrt{2}V_{r}sin\left(2\pi f_{n}t-\frac{2\pi }{3}\right)$$

The voltage applied to the VT/LPVT under test was composed of a fundamental tone (e.g., $v_{A}\left(t\right))$ plus 1 harmonic tone (FH1 test waveform) according to

(7) $$v_{\mathrm{FH}1}\left(t\right)=\sqrt{2}V_{r}sin\left(2\pi f_{n}t\right)+\sqrt{2}V_{h}sin\left(2\pi hf_{n}t\right)$$

where the test harmonics had amplitudes $V_{h}$ and orders *h* according to the values in Table 3.

- Proximity Effect

**Figure 7:**
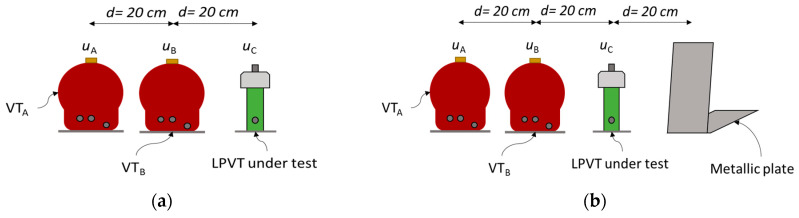
Proximity test arrangement to evaluate the impact of proximity on the VT/LPVT performance in harmonic measurements: (**a**) without the metallic plate (OOX) and (**b**) with the metallic plate (OOX|).

The same arrangements as those shown in Figure 6 for the LPVT were considered for the VT/LPVT proximity evaluation tests (Figure 7a). In addition, a metallic structure was placed at 20 cm from the VT/LPVT under test (Figure 7b). The presence of the metallic plate is indicated with the symbol “|”. The two adjacent phases were grounded (in-phase configuration), so that no electric field was generated by the adjacent phases.

- Combined Effect of Adjacent Phases and Proximity

The VT/LPVT in the external arrangement (OOX) was energized with the FH1 test waveforms. Additionally, a metallic plate was positioned in close proximity to the VT/LPVT at a distance of 20 cm (Figure 7b). The phases A and B were energized as outlined for the adjacent phase tests.

### 2.4. Effects of Adjacent Phase and Proximity on LPCTs and CTs

Two inductive CTs and one Rogowski coil (RC) were examined by PTB to determine the influence of the position and adjacent phases on their accuracy performance at 50 Hz. The measurement procedures and setups are described in [16]. The RC was tested using a self-developed integrator [16] that provided two output voltage ranges: $U_{\mathrm{out}}=0.25\;V$ and $U_{\mathrm{out}}=2.75\;V$. The rated characteristics of the DUTs are summarized in Table 4.

**Figure 8:**
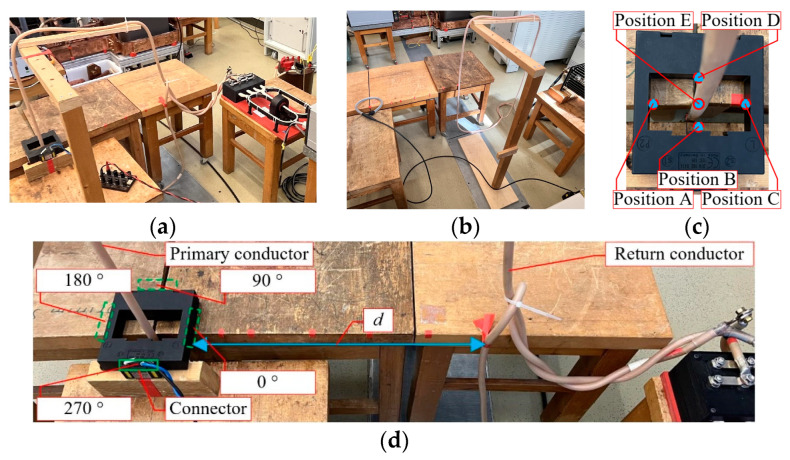
Photos of laboratory test setups for the considered influence quantities: (**a**) measurement setup for position effect, (**b**) measurement setup for adjacent effect, (**c**) position of centering, (**d**) return conductor effect as a function of the CT connector position.

The tests of the position effect were performed on CT II and CT III. The PTB laboratory measurement setup is presented in Figure 8a. The tests dealt with four aspects:

1. Primary conductor centering;
2. The position of the CT connector;
3. The distance *d* between the primary conductor through the DUT and its return conductor;
4. Test repeatability/random.

The tests of the adjacent phases were performed on all three current sensors, including the three aspects (2), (3), and (4). Photos of the laboratory measurement setup and details are presented in Figure 8, including (a) the measurement setup for the position effect, (b) the measurement setup for the adjacent effect, (c) the position of centering, and (d) the CT connector position, primary conductor, and return conductor.

For the centering test, as depicted in Figure 8c, measurements were conducted at five positions (A, B, C, D, E) in the primary conductor of the CTs (CT I and CT II). For CT III, various positions were tested to find the worst-case position.

For the connector position and distance tests, the laboratory measurement setup shown in Figure 8d involved testing at four positions (0°, 90°, 180°, 270°) for the connector placement. The distance *d* between the primary conductor through the DUT and its return conductor was set between 5 cm and 50 cm. In all tests, the primary current *I*_P_ was set to a sinusoidal waveform at power frequency with a current of 100 A.

The default setup parameters were as follows: primary conductor at position A; connector position at 270°; *d* = 50 cm. To validate the repeatability of the measurements, at least 7 measurements were repeated with the default setups for all DUT CTs. Additionally, the connector of CT III was opened and closed for each measurement repetition.

### 2.5. Adjacent Phases and Proximity Effect on LP Combined Sensors

#### 2.5.1. Generation and Measurement Setup

**Figure 9:**
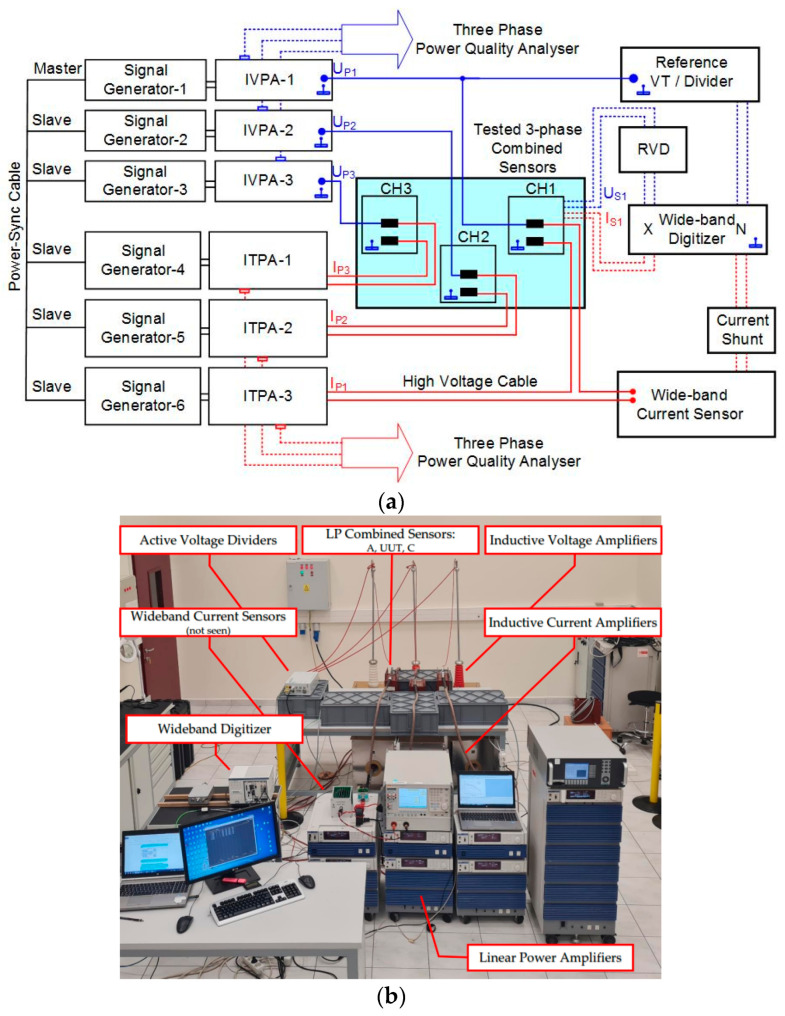
Generation and measurement setup for the assessment of adjacent phases and proximity effects on combined ITs and LP combined sensors’ accuracy in PQ measurement. (**a**) Schematic representation and (**b**) picture of the setup arranged in TÜBİTAK Power & Energy laboratory.

The three-phase measurement setup for the PQ characterization of MV combined ITs and sensors developed by TÜBİTAK is illustrated in Figure 9. The setup could generate voltages up to 36 kV and currents up to 2 kA; the amplitude and phase resolutions were 0.01% and 0.01° when setting the phase difference between them and between each phase. The generation part of the setup included 6-channel signal generators, the inductive voltage power amplifiers (IVPAs), and the inductive transconductance power amplifiers (ITPAs), while the measuring part of the setup consisted of a reference voltage divider, a reference current sensor, and a wideband digitizer. The LV signals produced by the signal generators were amplified by the IVPAs. The high voltages at the outputs of the IVPAs were applied to the LP combined sensors under test and to the reference voltage divider. The LV outputs of both the LP combined sensors under test and the reference voltage divider were compared by the wideband digitizer.

Similarly, for the high current generation, the high currents were generated by the ITPAs and then applied to both the LP combined sensors under test and the reference current sensor in series. The LV outputs of both the LP combined sensors under test and the reference current sensor were compared by the wideband digitizer. The ratio error and phase displacement of the LP combined sensors under test were extracted from the synchronized sampled voltage waveforms using the Fast Fourier Transform.

The reference voltage divider had an active unity-gain wideband buffer amplifier to enable a load-independent output and very small ratio and phase errors up to 9 kHz. This is required not only for impedance matching, but also for the elimination of the capacitive load of the digitizer inputs. A wideband fluxgate current sensor along with a wideband current shunt were used as a reference current sensor with a very small ratio and phase errors up to 9 kHz. The wideband digitizer, which was configured as a wideband bridge, was a 24-bit two-channel digitizer with a sampling rate of 204.8 kSa/s. This comprehensive setup enabled the analysis of multiple external magnetic and electrical fields simultaneously on each voltage and current measuring part of the three-phase combined ITs and sensors.

#### 2.5.2. Test Parameters and Procedures

Testing was focused on a Rogowki coil (RC) of an LP voltage and current combined sensor, along with two additional identical sensors. The RCs had a nominal primary current range of 1600 A, a nominal output voltage of 0.150 V (at 50 Hz), and a 0.5 accuracy class. The arrangements for the tested LP combined sensor are given in Table 5.

The central axes of the three sensors were maintained at a fixed distance of 20 cm from each other. Similarly, the primary current conductor of the tested sensor was vertically aligned at 20 cm from the central axes. The three supply currents were digitally synthesized to investigate the influence of the magnetic field produced by adjacent phases in two different scenarios: (i) when an in-phase power supply is present, and (ii) when a three-phase supply is active.

To assess the effects of the adjacent phases and proximity as individual and combined quantities impacting the performance of the RC sensor in harmonic measurements, three tests were carried out. Two tests were dedicated to evaluating the impact of each influence quantity independently, while one test was performed to assess the combined effect of both quantities. The fundamental tone, operating at the power frequency, had an amplitude $I_{r}=1600$ A, whereas the harmonics had amplitudes and orders as listed in Table 3. The two adjacent phases were energized with sinusoidal currents at 50 Hz and 1600 A. Both central and external arrangements, as well as the two current supply conditions, were considered in the tests.

## 3. Experimental Results: Effect of Single Influence Quantity

Experimental results obtained under a single influence quantity are synthesized in the following.

### 3.1. Temperature (VTs)

**Figure 10:**
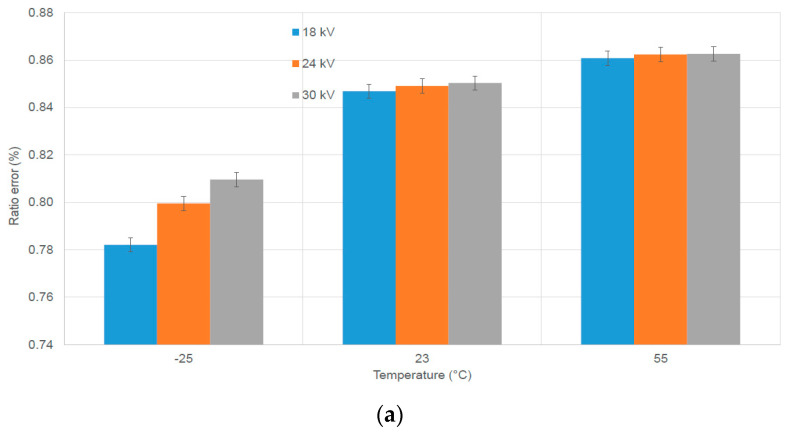

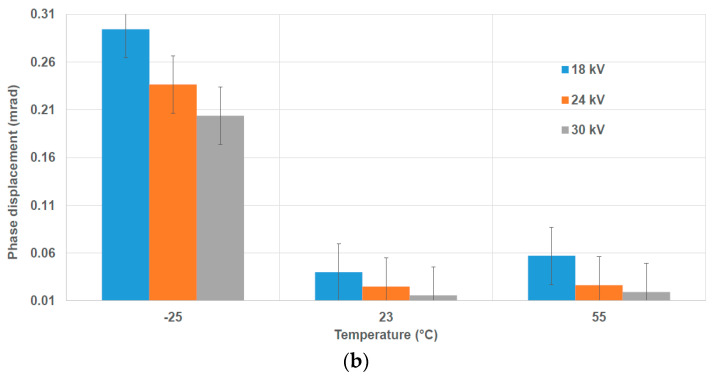
Power frequency errors vs. temperature for the investigated VT. (**a**) Ratio error; (**b**) phase displacement.

The values of the ratio error and phase displacement obtained at −55 °C, 23 °C, and 55 °C on the VT are presented in Figure 10 for the tests performed as described in Section 2.1. The results refer to the impact of temperature on the VT ratio error and phase displacement when the VT is supplied at a fundamental frequency, applying different primary voltages. The temperature has the most significant influence on the VT performance at −25 °C, showing variations of approximately 600 µV/V in the ratio error and approximately 250 µrad in phase displacement compared to the ambient temperature (23 °C). Notably, the effect of the applied voltage is more marked at very low temperatures of −25 °C.

Specifically, for the ratio error, we observed a voltage deviation of approximately 275 µV/V at −25 °C and less than 30 µV/V at 23 °C and 55 °C. Concerning phase displacement, the voltage deviation was approximately 90 µrad at −25 °C and less than 40 µrad at 23 °C and 55 °C. These variations in temperature may be attributed to the magnetic properties of the materials used in constructing the VTs. Despite these findings, it is important to note that the DUT remained within its declared accuracy class (1%).

As for the results obtained over the whole frequency range, the temperature has an impact on the frequency response, with the strongest influence at the resonance frequencies. The change in the temperature shifts the resonance frequencies as the winding geometry changes: with rising temperature, the resonance frequency decreases. Therefore, in the region close to the resonances, the ratio error for a given frequency can change in the range of 100% [31].

### 3.2. Vibration (VT)

Numerous measurements were conducted at 23 °C to investigate the potential impact of vibrations on the accuracy of the same inductive VT. Various parameters (rated voltage, axis of vibration, vibration frequency, and acceleration) were considered in combination. Although very slight deviations were observed, they fell within the range of measurement uncertainty (approximately 30 µV/V for ratio error and 30 µrad for phase displacement). Consequently, at this level of uncertainty, it was not possible to detect any significant influence of vibration on the VT performance at 50 Hz. Similarly, the investigation of the VT frequency response at 23 °C, up to 4 kHz, did not reveal a significative vibration influence.

### 3.3. Burden (VT)

The burden has a strong influence on the VT ratio error over the whole frequency range, the largest influence can be observed in the range of the resonance that is damped by the burden. At the fundamental level, the ratio error changes by up to 2%, whereas at the resonance, the ratio error changes by up to 120% [30].

### 3.4. Adjacent Phases

#### 3.4.1. VT/LPVTs

**Figure 11:**
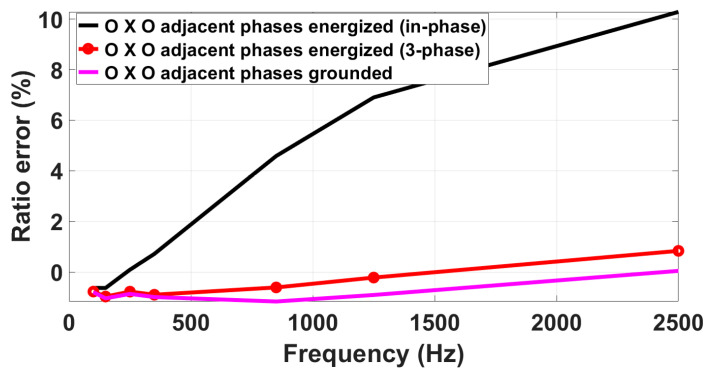
Impact of adjacent phases on the R-LPVT frequency response.

The first analysis focuses on examining how adjacent phases affect the harmonics responses of VT/LPVTs. Specifically, the results here reported consider the central position (OXO) case and three different supply conditions: in-phase (Equation (3)), three-phase (Equations (4)–(6)), and grounded adjacent phases. For brevity, only results related to the R-LPVT are presented in Figure 11. As a main result, it is found that the adjacent phases’ presence has an impact on the R-LPVT harmonics performance that increases with the increase in harmonic order and depends on the supply configuration. When examining the harmonic ratio error curve under a three-phase supply condition, not surprisingly, a behavior quite similar to that measured under grounded adjacent phases is found. At 100 Hz, the deviation between the two curves is 200 µV/V, increasing to 0.8% at 2500 Hz. On the other hand, the black curve, which represents the results under an in-phase supply condition, differs significantly from the case where the adjacent phases are grounded. At 100 Hz, the deviation between these two cases is 0.17%, while, at 2500 Hz, it exceeds 10%.

#### 3.4.2. LP Combined Sensors (RCs)

**Figure 12:**
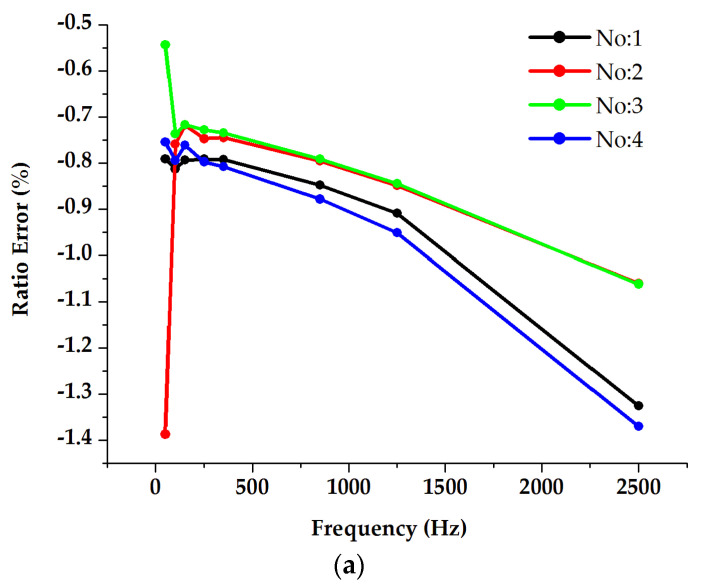

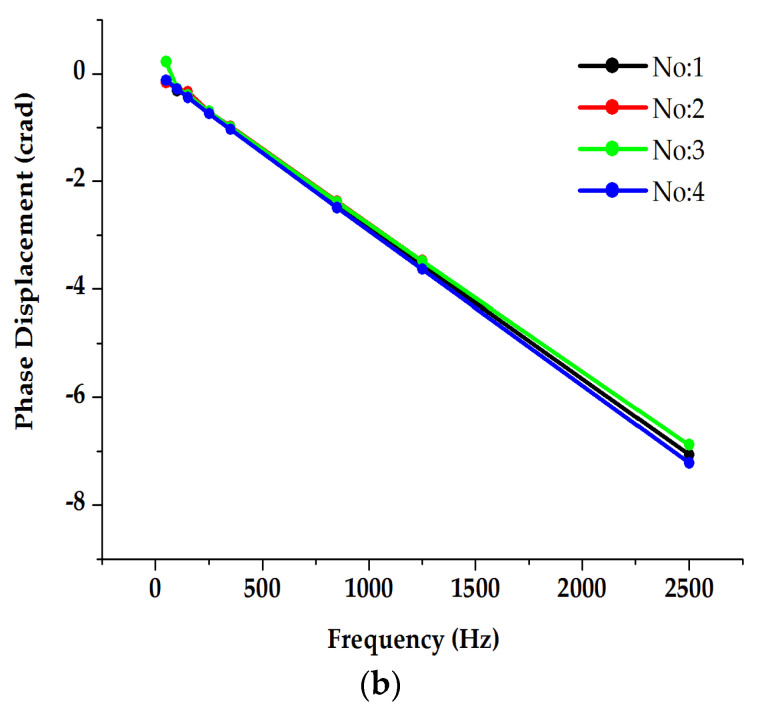
Analysis of the impact of current supplied adjacent phases on the combined current sensor (RC) frequency response for both ratio error (**a**) and phase displacement (**b**).

The main objective of this test was to investigate the potential influences on the ratio errors and phase displacements when considering three different arrangements of adjacent phases and comparing them with stand-alone measurements. All results are shown in Figure 12. The stand-alone frequency response measurement results (No. 1) indicate that the DUT exhibits ratio errors with a deviation of −0.6% up to 2500 Hz with respect to the 50 Hz error, whereas the phase displacements show a significant difference of up to several centiradians. The in-phase and three-phase supply conditions (No. 2 and No. 3) show similar effects for the ratio errors at frequencies above 50 Hz. As for the power frequency ratio errors, higher deviations are found due to the presence of high currents, leading to elevated external magnetic fields. Moreover, when relocating the DUT to another phase position away from the center (No. 4), the study revealed relatively regular behavior of the ratio error deviation, particularly for frequencies above low-frequency harmonics. A significant difference was detected instead for the phase displacement.

### 3.5. Proximity

#### 3.5.1. VT/LPVTs

**Figure 13:**
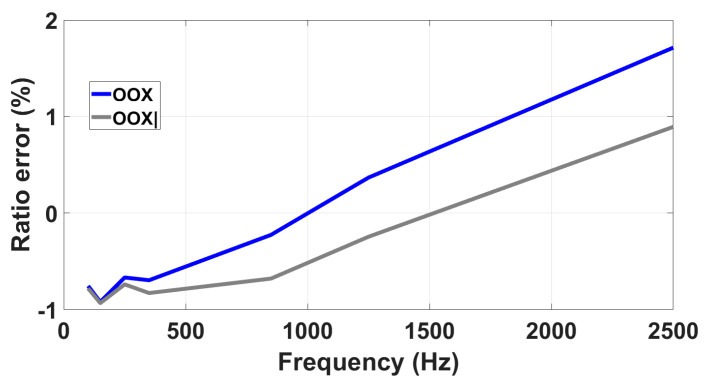
Analysis of the impact of proximity on R-LPVT frequency response.

The impact of the proximity effect was verified by positioning the DUT in an external arrangement and grounding the adjacent phases, so that their effects in terms of the generated electric field could be disregarded. Referring to the R-LPVT tests, Figure 13 presents two distinct curves: one obtained with the R-LPVT in the external position (OOX), and the other with the R-LPVT in the external position with the metallic plate at a distance *d* = 20 cm (OOX|). It is evident that the tested R-LPVT is sensitive to the proximity effects and the difference between the two curves becomes more pronounced as the frequency increases: at 100 Hz, the deviation is 0.03%, whereas at 2500 Hz, it reaches 0.80%.

#### 3.5.2. LP Combined Sensors (RCs)

**Figure 14:**
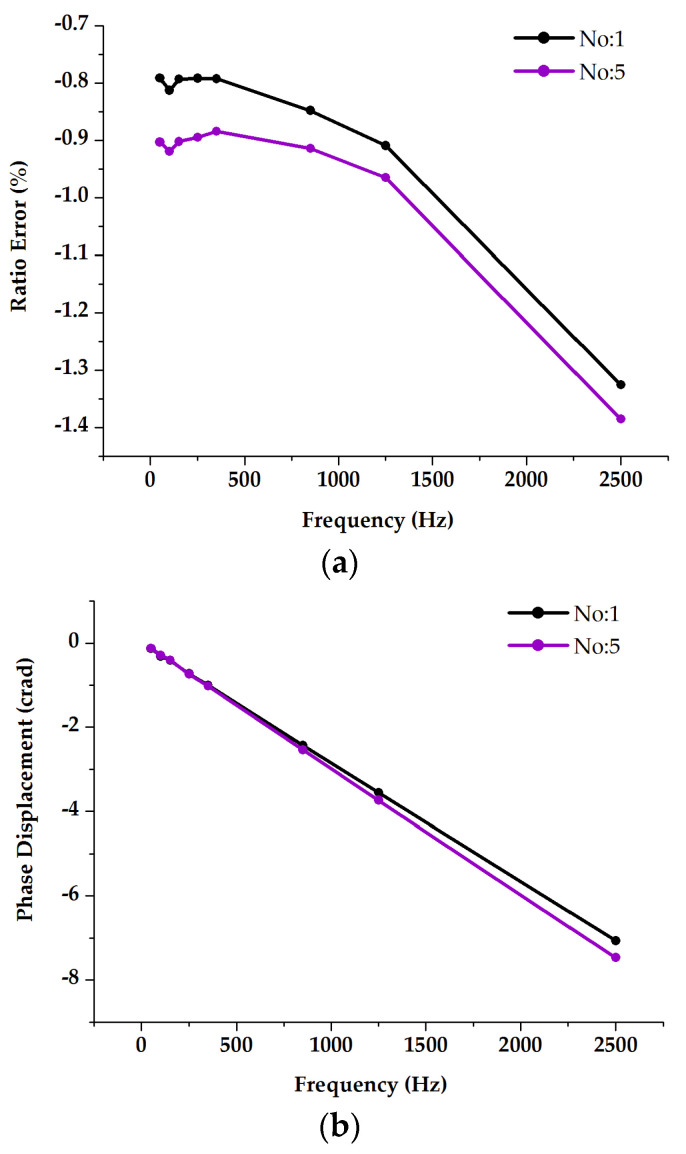
Analysis of the impact of proximity on combined sensor (RC) frequency response for both ratio error (**a**) and phase displacement (**b**).

The primary objective of this test was to investigate the influences of proximity effects on the RC ratio errors and phase displacements in the harmonic range, by altering the alignment of the primary carrying conductor. The obtained results are shown in Figure 14 (No. 5). The effect, with respect to the tests carried out on the single RC, is detectable, but not so significant. From this point of view, it is worth considering the RC as a closed loop one.

## 4. Experimental Results: Effect of Multiple Influence Quantities

This section provides experimental results measured under combined influence quantities.

### 4.1. Temperature and Vibration

**Figure 15:**
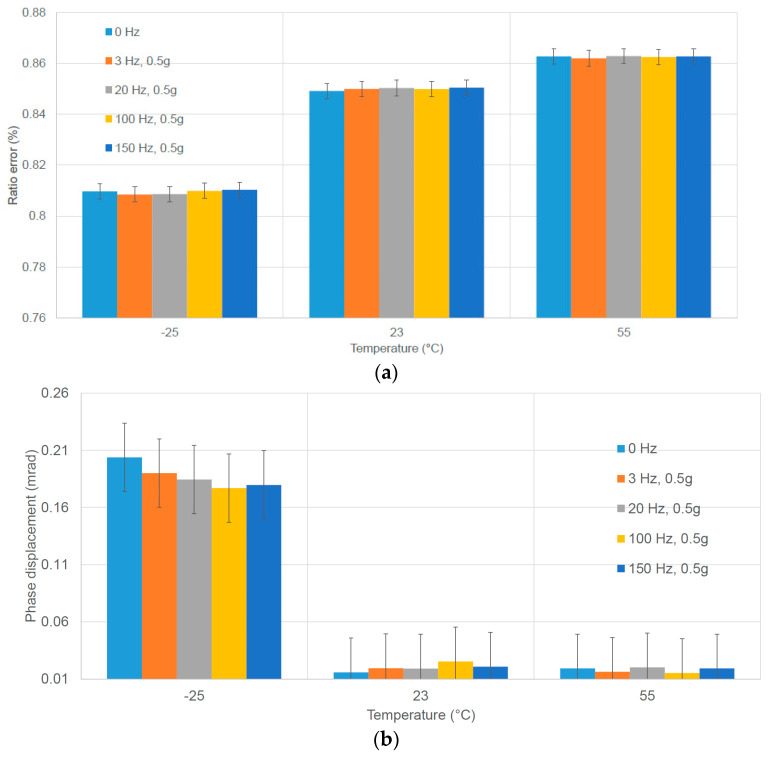
Accuracy performance in terms of ratio errors and phase displacement at power frequency vs. temperature (−25 °C, 23 °C, and 55 °C) and vibration (from 0 Hz, without vibration, to 150 Hz) for the VT. (**a**) Ratio error; (**b**) phase displacement.

The combined influence of temperature and vertical vibration can be analyzed by looking at Figure 15. The measurements were conducted under a sinusoidal condition at 30 kV; with the shaker configured for vertical vibration at acceleration of 0.5 g, the investigated vibration frequencies ranged from 0 Hz (without vibration) to 150 Hz. The results, in terms of the ratio error and phase displacement at the fundamental frequency, are reported for three selected temperatures: −25 °C, 23 °C, and 55 °C.

Notably, the most significant deviation is evident at −25 °C. However, the results, both with and without vibration, at each temperature, demonstrate only minimal deviations. Not surprisingly, when both parameters, temperature and vibration, were applied simultaneously, the effects were not more pronounced than when they were applied separately.

The frequency response of the VT was measured under the combined influence of temperature and vibration when harmonics were added. For each temperature level, the initial measurement was performed without vibrations or harmonics, serving as the reference for subsequent comparisons. Subsequently, the shaker was set to generate vertical vibrations at 0.5 g (m/s^2^) with frequencies of 20 Hz and 100 Hz. Using these settings, several measurements were conducted with an applied voltage of 30 kV and a frequency of 50 Hz, including harmonics ranging from 150 Hz up to 4000 Hz. The amplitude of these harmonics was maintained at 5% (or 3% above 2 kHz) of the fundamental frequency. When examining the accuracy at 50 Hz, the maximum variation in the ratio error was less than 30 µV/V, and the phase displacement exhibited less than 30 µrad. These variations remained within the VT’s accuracy class, and they could be attributed to the uncertainty of the measurement, which fell within the same range. Interestingly, the presence of harmonics and vibration did not significantly influence the results when the temperature was kept constant. Similar observations were made when measuring the frequency response of the VT. However, at low temperatures, the deviations were more noticeable, regardless of the presence of vibration [31,40].

### 4.2. Temperature and Burden

Regarding the impact of the burden with different temperatures, it is observed that the impact of the VT burden decreases nonlinearly with a rising temperature. At a fundamental frequency, when comparing the ratio errors at the rated burden with respect to errors at no burden for different temperatures, it is found that the ratio error changes by 4.5% at −25 °C, by 2% at 20 °C, and by 1.5% at 55 °C. The same trend, with larger changes, occurs in the higher harmonics range [40].

### 4.3. Adjacent Phases and Proximity

#### 4.3.1. LPVTs and VTs

The results of the tests carried out on the VT/LPVTs listed in Table 2 are shown in this subsection. They refer to the case of the in-phase VT supply and external positioning (OOX) condition. For each tested transducer, a related diagram with three ratio error curves is shown: two curves were obtained in the presence of a single influence quantity (adjacent phases and proximity), and the third one provides the results under the combined effect.

- R-LPVT

**Figure 16:**
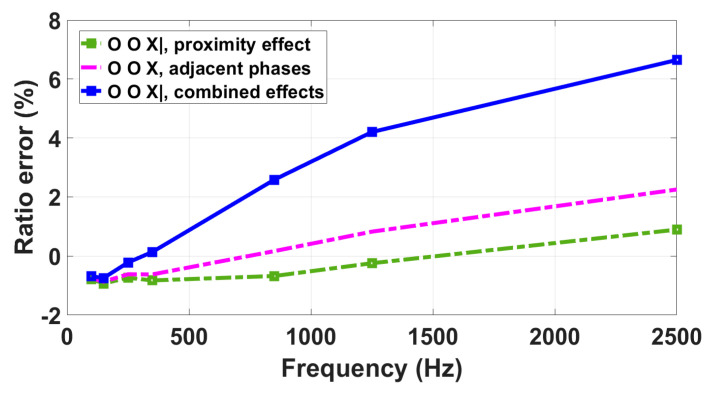
Analysis of the impact of proximity, adjacent phases, and their combined effects on the R-LPVT frequency response.

The results of the tests conducted on the R-LPVT are presented in Figure 16 and summarized in Table 6, where *ε*_adjacent-phases_ is the ratio error measured in the presence of the supplied adjacent phases as a single influence quantity, *ε*_prox_ is the one measured under the proximity effect only, and *ε*_comb_ is the ratio error measured under combined influence quantities. It can be noticed that the ratio errors measured under the three different conditions exhibit quite small changes for the first two harmonics, with deviations within 0.2%. However, as the frequency increases, the deviations between the combined effect curve and the single influence quantity curves start to significantly increase.

In particular, at 2500 Hz, the ratio error measured under the proximity effect is equal to 0.90%, the one related to the adjacent phase effect is 2.25%, and that measured under combined effects is equal to 6.65%. Consequently, the combined presence of the two influence quantities worsens the frequency response. With the error measured in the presence of combined effects being significantly higher than the sum of the single influence quantity errors, it is clear that it cannot be evaluated only by single quantity tests.

- C-LPVT

**Figure 17:**
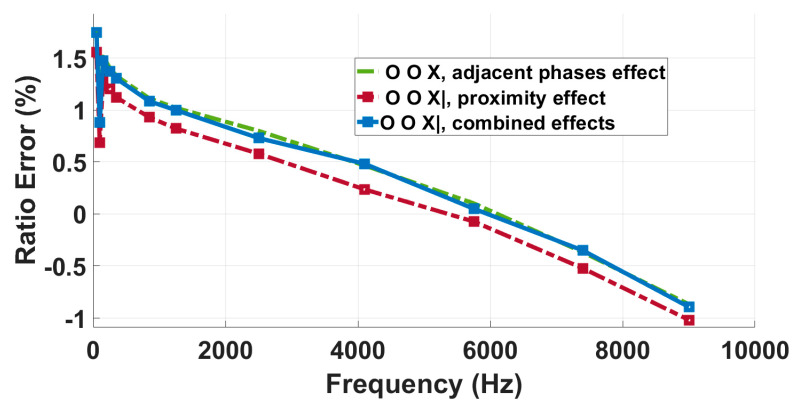
Analysis of the impact of proximity, adjacent phases, and their combined effects on the C-LPVT frequency response.

From Figure 17 and Table 7, it can be seen that the frequency responses obtained for the tested C-LPVT under the combination of influence quantities and the one from the adjacent phase test overlap, over the entire analyzed frequency range (up to 9 kHz), when the in-phase supply configuration is used. The frequency response measured in the presence of the metallic plate with the adjacent phases grounded differs from the combined effect curve by a nearly constant value of 0.2% from 50 Hz to 9 kHz.

Based on these findings, it can be concluded that the adjacent phases’ influence is the predominant one, whereas the proximity due to the external metallic plate is quite negligible for the tested C-LPVT.

- RC-LPVT

**Figure 18:**
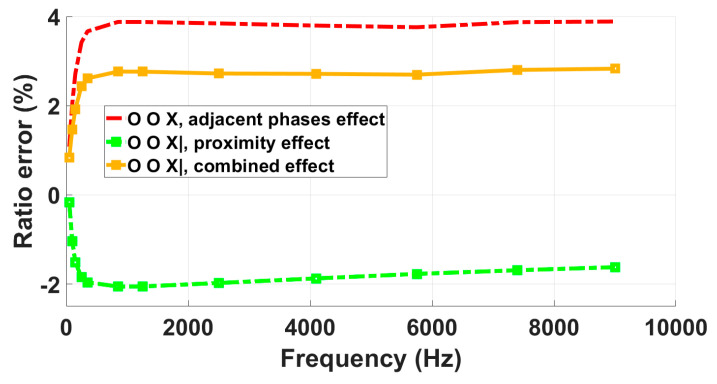
Analysis of the impact of proximity, adjacent phases, and their combined effects on RC-LPVT response up to 9 kHz.

Differing from the C-LPVT, the frequency performance of the tested RC-LPVT was highly sensitive to the investigated influence quantities, as evidenced by the variations in the three curves in Figure 18, which differ by a few to several percent depending on the test conditions up to 9 kHz. As evidenced in Table 8, for this DUT, the combined effect results can be approximated at least up to 9 kHz by the sum of the single influence quantity errors, with a maximum deviation between the summed and experimentally measured values in the combined test of less than 1%.

- VT

**Figure 19:**
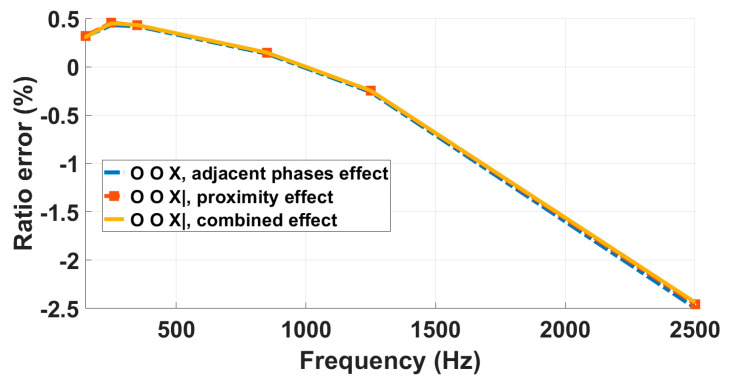
Analysis of the impact of proximity, adjacent phases, and their combined effects on VT frequency response.

The results summarized in Figure 19 and numerically quantified in Table 9 indicate that the tested inductive VT was quite immune to both the adjacent phases and proximity effects, as the three curves overlapped, with a maximum ratio error difference among them of 0.05%.

#### 4.3.2. LPCTs and CTs

The maximum differences in the DUTs’ errors at 50 Hz, with a nominal burden, in various tests for position effects are listed in Table 10. The centering of the primary conductor had the dominant influence on the position effects.

To test the adjacent effects without using a three-phase current system, the DUTs were tested in the absence of a primary conductor and with a 100 A primary input for the reference CT (Figure 8b). In this case, the measured DUT errors *E*_X_ can be considered to be caused by adjacent return effects. Additionally, the evaluation was first focused on the ratio errors. Finally, the obtained ratio errors *E* were applied to all angles, *E* = *E*_X_/*U*_N,S-rated_·*U*_X,S-rated_, where *U*_N,S-rated_ and *U*_X,S-rated_, respectively, represent the rated secondary voltages of the reference CT and the DUT CT. As a result, the maximum difference in the ratio errors Δ|*E*| or the maximum ratio errors |*E*|_max_ at 50 Hz with a nominal burden in various tests of the adjacent effects are as summarized in Table 11. The connector position of the primary conductor had the dominant influence on the adjacent effects.

#### 4.3.3. LP Combined Sensors (RCs)

**Figure 20:**
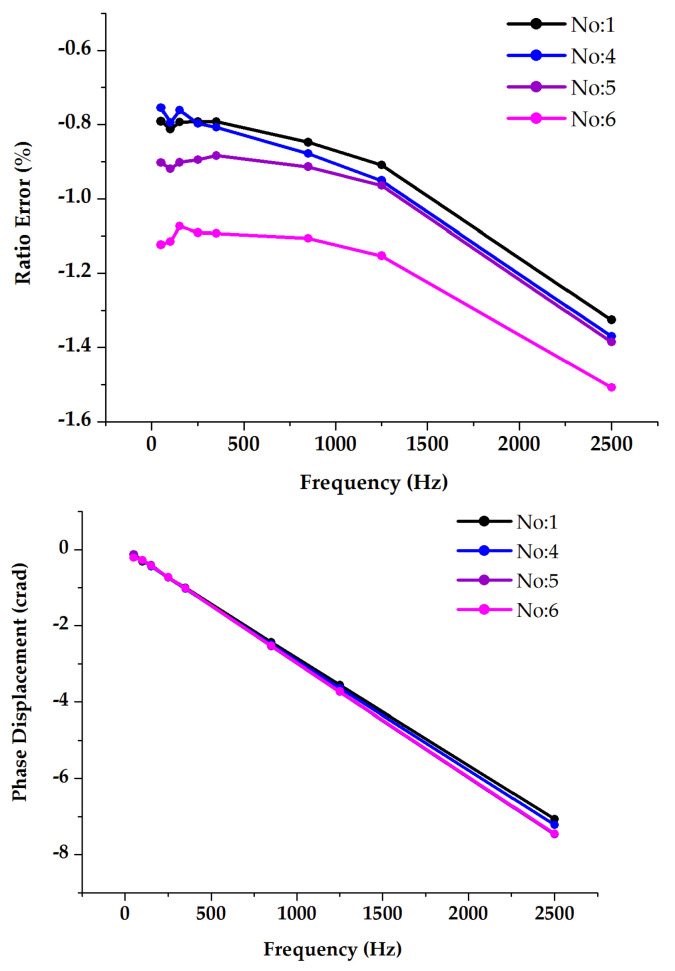
Analysis of the impact of adjacent phases, proximity, and their combined effects on combined sensor (Rogowski) frequency response for both ratio error and phase displacement.

This test aimed to investigate the influence on the ratio errors and phase displacements resulting from the combined arrangement of the No. 4 and 5 supply conditions; see Figure 20. The combined effects (No. 6) on the ratio error deviations clearly indicate that the combination of adjacent phases and proximity effects is consistently larger than their individual influences from 50 Hz up to 2500 Hz. The effect is less evident but still present for the phase.

## 5. Discussion of the Experimental Results

This section provides an overview of the main findings related to the considered DUTs and their associated influence quantities. For all the cases, the analysis is focused on the ratio error variation over the investigated frequency range. To facilitate a clearer understanding, the results are summarized in three tables (Table 12, Table 13 and Table 14) that categorize the impact of the influence quantities into three different groups: low (variation < 1%), medium (variation between 1% and 3%), and high (variation > 3%). The analysis provided in the tables introduced in this section help to compare the impacts of the different influence quantities on the different types of investigated ITs. In fact, the same thresholds of 1% and 3% were consistently applied to all cases, enabling a comprehensive comparison of the results derived from the extensive experimental activity.

### 5.1. Temperature and Vibration

The results of the VT characterization with temperature and vibration as influence quantities indicate that the temperature is the parameter with the strongest influence on the studied inductive VT’s performance. Low temperatures, such as −25 °C, have the highest impact on the ratio error and the phase displacement from 50 Hz up to 4 kHz.

The mechanical external vibrations combined or not with temperature introduce variations comparable with the measurement uncertainty, so they can be considered as negligible influence quantities (Table 12).

### 5.2. Temperature and Burden

Based on the experimental results, the burden has a significant impact on the ratio error in the whole frequency range. Without the burden, the temperature has a significant impact on the resonance frequencies; therefore, the ratio error changes the most as the frequency approaches the first resonance frequency. At lower frequencies, the influence becomes low (Table 12).

As for the combined burden and temperature, the temperature influences the impact of the burden nonlinearly. Compared to 20 °C, a change to 55 °C causes only a small change in the burden dependency of the ratio error, while a change to −25 °C causes a large difference in the burden dependency of the ratio error (Table 12).

### 5.3. Adjacent Phases and Proximity

#### 5.3.1. VTs and LPVTs

Devices operating based on different principles were tested. Sensor positioning (central or external) and the voltage supply of adjacent phases (in-phase or three-phase) were demonstrated to impact the results. A three-phase supply, with respect to adopting an in-phase voltage test, provided results closer to those obtained with grounded adjacent phases, because of the partial compensation of the generated three-phase power frequency electric fields.

The main findings indicate that the considered influence quantities have different impacts on the tested devices. Therefore, it is not possible to draw general conclusions. Considering the single type of sensor investigated, the results are summarized in Table 13.

#### 5.3.2. CTs and LPCTs

During the testing of the current sensors, two inductive CTs and one Rogowski coil with a self-developed integrator were evaluated for their susceptibility to position and adjacent effects at power frequency. Based on the experimental results, both the inductive CT and LPCT were found to be significantly affected by the centering of the primary conductor in terms of position effects, while the position of the connector of the primary conductor had a major impact on adjacent effects. Specifically, in the case of the inductive CT, both the centering and the connector position of the primary conductor exhibited nearly equal influences with a relatively low impact. On the other hand, in the case of the LPCT, the maximum error difference resulting from the centering influence of the primary conductor was approximately 1%, which was approximately 10 times larger than the maximum error difference caused by the connector position influence of the primary conductor; see Table 14.

#### 5.3.3. LP Combined CT Sensors

Based on the experimental results, the RC of the tested combined sensor was found to be affected in terms of both the adjacent phase and proximity, with certain differences for almost every arrangement, as follows.

The influences on the ratio errors and phase displacements of the in-phase and three-phase arrangement were measured with a similar approach, when the tested sensor was placed in the middle. In this case, power frequency errors were significantly affected due to the adjacent phases’ 50 Hz current.

The adjacent phase effects on the ratio errors were found closer to the stand-alone results when changing its position from the middle.

Proximity tests that involved changing the alignment of the primary current carrying conductor showed that the influences on the phase displacement increased as a function of the frequency. On the other hand, this consideration was not valid for the ratio errors.

It was found that the combined influences on the ratio error results were greater than the individual influences from the adjacent phases and proximity. A summary of the main findings is included in Table 14.

## 6. Conclusions

Measurement setups and procedures that ensure the accuracy and traceability of the measurements carried out for the frequency characterization of MV voltage and current sensors with different operating principles under different influence quantities have been presented. These reference measurement and generation setups, developed within a collaborative effort by several national metrology institutes, can be used as a basis to develop simpler but still accurate systems to be used in manufacturers’ or users’ laboratories.

The results of an extensive measurement investigation carried out to both identify and quantify the most critical influence quantities have been discussed, delineating their most detrimental combinations and highlighting those quantities that do not significantly impact the frequency performance of ITs.

The experimental activities have highlighted that the temperature has a significant impact on the performance of inductive VTs, particularly at low temperatures, resulting in remarkable changes in ratio error and phase displacement, especially near the resonance frequency. Mechanical vibrations have a negligible effect, even in combination with temperature variations. The VT burden significantly influences the ratio error, with its impact intensifying as the temperature decreases and frequency increases. The proximity and adjacent phase parameters have clearly no impact on VT performance. As for LPVTs, the impact of the adjacent phases and proximity varies for different types of voltage LPVTs, making it challenging to draw general conclusions. For CTs and LPCTs, the centering of the primary conductor and the position of the return connectors show the strongest influence. As for the interactions between two influence quantities and their combined impact on the frequency performance of ITs, they are not easily predictable a priori, being strongly dependent on the considered ITs, in terms of both the operating principles and construction solutions adopted.

## Figures and Tables

**Table 1:** Characteristics of the tested VTs.

Name and photo of the investigated VT		**VT A**	**VT B**	**VT C**	**VT D**
		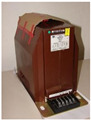	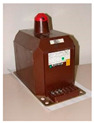	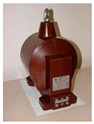	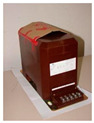
Rated primary voltage	(kV)	20/√3	20/√3	35/√3	10/√3
Rated secondary voltage	(V)	100/√3	100/√3	100/√3	100/√3
Rated frequency	(Hz)	50	50	50	50
Rated burden	(VA)	50	50	50	30
Accuracy class		0.5	0.5	0.5	0.5
Thermal time constant	(h)	2.4	2.2	2.1	1.6

**Table 2:** Rated characteristics of the tested VT/LPVTs.

Name of the DUT	Type	Primary Voltage (kV)	Rated Scale Factor (V/V)	Accuracy Class
R-LPVT	Resistive divider	20/√3	6153.8	0.5
C-LPVT	Capacitive divider	20/√3	20/√3	0.5
RC-LPVT	Resistive–capacitive divider	45	10,000	0.2
VT	Inductive VT	20/√3	200	0.5

**Table 3:** FH1 test—harmonic characteristics.

h (-)	$V_{h}$ (%)
2	2
3	5
5	6
7	5
17	2
25	2
50	0.5
82	0.5
115	0.5
148	0.5
180	0.5

**Table 4:** Rated characteristics of the investigated current sensors.

Main Features of the DUT	Inductive CT		Rogowski Coil
Name	CT I	CT II	CT III
Primary current	400 A	500 A	1000 A
Output	1 A	1 A	22.5 mV
Transformation ratio	1:400 A/A	1:500 A/A	-
Rated burden	5 VA	2.5 VA	10 kΩ
Accuracy class	Cl. 0.2S	Cl. 0.5	Cl. 0.5

**Table 5:** Positioning details for the tests performed on the LP combined sensors.

1	UUT-LP Combined Sensor (RC): Sensor UUT is tested as a standalone device by applying a multitone (FH1) test signal.	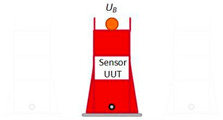
2	Adjacent Phases (in-phase supply): Sensor UUT is placed in the middle (B) between sensors A and C, which are energized with the same fundamental tone as Sensor UUT, but without harmonics.	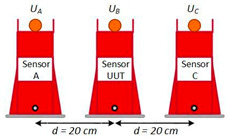
3	Adjacent Phases (three-phase supply): Sensors A and C are energized with ±120° phase differences from UUT.	
4	Adjacent Phases (in-phase supply): Sensor UUT is replaced by Sensor C, while Sensors A and C are energized with the same phase as Sensor UUT	
5	Proximity (return cable alignment): The primary current conductor is aligned 90° downwards with a distance of 20 cm from the central axes.	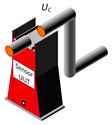
6	Combined (adjacent phases and proximity): Arrangements 4 and 5 are placed together. (Figure is for demonstration only). Sensors are aligned as in previous arrangements.	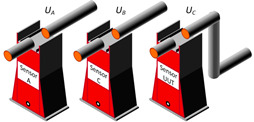

**Table 6:** Ratio error for the combined tests on the R-LPVT.

Frequency (Hz)	*ε*_adjacent-phases_ (%)	*ε*_prox_ (%)	*ε*_comb_ (%)
150	−0.87	−0.93	−0.74
850	0.16	−0.68	2.58
1250	0.82	−0.24	4.20
2500	2.25	0.90	6.65

**Table 7:** Ratio error for the combined tests on the C-LPVT.

Frequency (Hz)	*ε*_adjacent-phases_ (%)	*ε*_prox_ (%)	*ε*_comb_ (%)
100	0.92	0.69	0.88
350	1.33	1.12	1.30
1250	1.02	0.82	1.00
9000	−0.87	−1.02	−0.89

**Table 8:** Ratio error for the combined tests on the RC-LPVT.

Frequency (Hz)	*ε*_adjacent-phases_ (%)	*ε*_prox_ (%)	ε_adjacent-phases_ + ε_prox_ (%)	*ε*_comb_ (%)	*ε*_comb-_ (*ε*_adjacent-phases_ + *ε*_prox_)
50	1.08	−0.17	0.91	0.84	−0.07
350	3.66	−1.96	1.70	2.60	0.90
9000	3.88	−1.62	2.26	2.83	0.57

**Table 9:** Ratio error for the combined tests on the VT.

Frequency (Hz)	*ε*_adjacent-phases_ (%)	*ε*_prox_ (%)	*ε*_comb_ (%)
150	0.31	0.32	0.31
850	0.13	0.15	0.15
1250	−0.26	−0.25	−0.24
2500	−2.49	−2.45	−2.44

**Table 10:** Maximum difference in ratio and phase errors at 50 Hz with rated burden in various tests for position effects (primary current *I*_P_ = 100 A).

Position	CT II		CT III	
	Δ*ε* (%)	Δ*φ* (crad)	Δ*ε* (%)	Δ*φ* (crad)
(1)	0.04	0.02	0.99	0.01
(2)	0.01	0	0.09	0
(3)	0.01	0	0.02	0
(4)	0.02	0.03	0.16	0

**Table 11:** Results at 50 Hz with rated burden in various tests for adjacent effects (*I*_P_ = 100 A).

	CT I	CT II	CT III (*U*_out_ = 0.25 V)	CT III (*U*_out_ = 2.75 V)
	Δ|*E*| in ppm	Δ|*E*| in %	Δ|*E*| in %	Δ|*E*| in %
(2)	8	0.03	0.19	0.15
(3)	1	0.01	0.01	0.01
	**|*E*|max in ppm**	**|*E*|max in %**	**|*E*|max in %**	**|*E*|max in %**
(4)	2	0.009	0.019	0.016

**Table 12:** Summary of the results obtained from the inductive VT testing vs. temperature and vibration and vs. temperature and burden.

DUT	Temperature	Vibration	Combined
**Inductive VT**	High impact, most critical situation at lowest temperature (−25 °C) and frequencies close to the resonance	No impact	No impact of vibration
		**Burden**	**Combined**
		High impact	Burden impact decreases with rising temperature, most critical situation at −25 °C

**Table 13:** Summary of the results obtained from the VT/LPVT testing vs. adjacent phases and proximity.

DUT	Adjacent Phases	Proximity	Combined
**R-LPVT**	Medium impact	Medium impact	High impact, Combined error higher than the sum of single influence parameter errors
**C-LPVT**	Low impact	No impact	No impact of proximity
**RC-LPVT**	Medium impact	High impact	High impact. Possible to assume a direct sum of the two influence quantities (error associated with the sum of the effects < 1% up to 9 kHz)
**Inductive VT**	No impact	No impact	No impact

**Table 14:** Summary of the results obtained from the CT/RC testing vs. adjacent phases and proximity.

DUT	Adjacent Phases	Proximity	Combined
**RC (alone and as part of combined sensor)**	High impact (for power frequency errors only)	Medium impact	Higher impact, error higher than the sum of the single influence parameters
**Inductive CT**	Low impact	Low impact	Medium impact

## Abbreviations

**Abbreviation/Acronym**	**Definition**
AC	Alternate Current
ADC	Analogue-to-Digital Converter
AWG:	Arbitrary Waveform Generator
C-LPVT	Capacitive Low-Power Voltage Transformer
CT	Current Transformer
DAC	Digital-to-Analogue Converter
DAQ	Data Acquisition
DUT	Device Under Test
FH1	Fundamental Tone Plus 1 Harmonic Tone Waveform
HV	High Voltage
IT	Instrument Transformer
ITPA	Inductive Transconductance Power Amplifier
IVPA	Inductive Voltage Power Amplifier
LP	Low Power
LPCT	Low-Power Current Transformer
LPVT	Low-Power Voltage Transformer
LV	Low Voltage
LVMS	Low-Voltage Measuring System
MV	Medium Voltage
NMI	National Metrology Institute
PI	Performance Index
PQ	Power Quality
PXI	PCI eXtension for Instrumentation
RC	Rogowski Coil
RCVD	Resistive–Capacitive Voltage Divider
R-LPVT	Resistive Low-Power Voltage Transformer
RC-LPVT	Resistive–Capacitive Low-Power Voltage Transformer
UUT	Unit Under Test
VT	Voltage Transformer
